# Soy Food Consumption, Exercise, and Body Mass Index and Osteoporotic Fracture Risk Among Breast Cancer Survivors: The Shanghai Breast Cancer Survival Study

**DOI:** 10.1093/jncics/pkz017

**Published:** 2019-05-21

**Authors:** Neil Zheng, Evelyn Hsieh, Hui Cai, Liang Shi, Kai Gu, Ying Zheng, Ping-Ping Bao, Xiao-Ou Shu

**Affiliations:** 1 Yale College, Yale University, New Haven, CT; 2 Section of Rheumatology, Yale School of Medicine, Yale University, New Haven, CT; 3 Division of Epidemiology, Department of Medicine, Vanderbilt-Ingram Cancer Center, Vanderbilt University Medical Center, Nashville, TN; 4 Shanghai Municipal Center for Disease Prevention and Control, Shanghai, China; 5 Shanghai Cancer Hospital, Fudan University, Shanghai, China

## Abstract

**Background:**

Breast cancer survivors have a high incidence of osteoporosis-related fractures; the associated factors are understudied. We investigated incidence of bone fracture and its associations with soy food consumption, exercise, and body mass index among breast cancer survivors.

**Methods:**

This prospective study included 4139 stage 0–III breast cancer patients and 1987 pre-/perimenopausal and 2152 postmenopausal patients. Fractures were assessed at 18 months and at 3, 5, and 10 years after cancer diagnosis. Osteoporotic fractures were defined as fractures caused by falls from standing height and at sites associated with osteoporosis. Exercise and soy isoflavone intake were assessed at 6 and 18 months postdiagnosis. Weight and height were measured at baseline. Lifetable and Cox regression analyses were employed. All statistical tests were two sided.

**Results:**

The 10-year incidence for osteoporotic fractures was 2.9% and 4.4% for pre-/perimenopausal and postmenopausal patients, respectively. High soy isoflavone intake was associated with reduced risk among pre-/perimenopausal patients (hazard ratio [HR] = 0.22, 95% confidence interval [CI] = 0.09 to 0.53, for soy isoflavone mg/d ≥56.06 vs <31.31; *P*_trend_ < .001) but not among postmenopausal patients (*P*_interaction_ < .01). Overweight (vs normal weight) was a risk factor for pre-/perimenopausal patients (HR = 1.81, 95% CI = 1.04 to 3.14) but not for postmenopausal patients (HR = 0.67, 95% CI = 0.43 to 1.03; *P*_interaction_ = .01). Exercise was inversely associated with osteoporotic fractures in postmenopausal patients (HR = 0.56, 95% CI = 0.33 to 0.97, for metabolic equivalents hours ≥12.6 vs <4.5) following a dose-response pattern (*P*_trend_ = .035), an association not modified by menopausal status.

**Conclusions:**

Our findings, especially the novel association of soy food intake with osteoporotic fractures in breast cancer survivors, if confirmed, can help guide future strategies for fracture risk reduction in this vulnerable population.

Breast cancer patients have a higher incidence of osteoporosis-related fractures compared with age-matched healthy women (1,2). This increased risk is largely attributed to the decrease in bone mineral density (BMD) that occurs as a result of therapies that induce estrogen deprivation (2), a well-established risk factor for osteoporosis and associated bone fracture (3). Such treatments include endocrine therapies, such as tamoxifen or aromatase inhibitors, which are common adjuvant treatments for hormone receptor-positive breast cancers, the most common type of breast cancer (3). In addition, chemotherapy or ablation of ovarian function (either medically or surgically) can lead to premature menopause among younger women and reduce BMD (4).

Estrogen exerts its effect on target cells through binding estrogen receptors (ERs) (3). Modulators of ERs thus play an important role in bone health. For example, tamoxifen, a common adjuvant therapy for ER-positive breast cancer, is a selective estrogen receptor modulator (SERM) that competes with endogenous estrogen binding to the ERs to exert an antagonistic or agonistic effect, depending on target tissue (5). Whereas it inhibits estrogen's effect on breast cancer cells, tamoxifen acts as a partial estrogen agonist in the bone (5), particularly among postmenopausal women whose endogenous estrogen level is low, leading to increases in BMD (5). Soy foods are rich in isoflavones, a class of natural SERM that has previously shown to be inversely associated with the risk of death and recurrence in breast cancer patients (6,7). Soy food consumption has also been associated with reduced risk of incident fracture among healthy postmenopausal women, particularly during early menopause (8). The influence of soy food intake on bone fracture among breast cancer survivors, however, has not been assessed.

Several other modifiable lifestyle-related factors may influence the risk of bone fracture via estrogen-related and other biological mechanisms (9). Body mass index (BMI) and exercise, both of which are associated with physical fitness and estrogen levels, have been investigated for associations with BMD and osteoporotic fracture risk in postmenopausal women (10). However, few of the previous studies were conducted among breast cancer patients (10–14). Finally, premenopausal breast cancer patients have been particularly underrepresented in prior studies on osteoporotic fracture.

In the present study, we investigated the incidence of clinical osteoporotic fracture among breast cancer survivors and evaluated its associations with soy food consumption, BMI, and exercise in a large prospective observational cohort of Chinese breast cancer survivors.

## Methods

This study used data from the Shanghai Breast Cancer Survival Study (SBCSS), a large population-based longitudinal cohort of 5042 breast cancer survivors. Details of the study design and methodology have been previously described (6). Briefly, 5042 patients with newly diagnosed breast cancer, aged 20 to 75 years, were identified from the Shanghai Cancer Registry between March 2002 and April 2006 and enrolled in the SBCSS approximately 6 months after cancer diagnosis. At study enrollment, detailed information regarding patient demographics, cancer diagnosis and treatment history, medication use, dietary habits, exercise, and other lifestyle factors was collected via an in-person interview. Cancer diagnosis and clinical information, including age at diagnosis, cancer stage, and treatment, were verified by a review of medical charts. In-person follow-up was conducted at 18 months and at 3, 5, and 10 years after diagnosis to collect information on cancer outcome and changes of health status and to update lifestyle and medication use information. Survival and disease status data were supplemented by regular record linkage with the Shanghai Vital Statistics Registry. At years 3, 5, and 10, self-reported data regarding bone fracture occurrence, including affected sites and possible causes, were collected. The response rates for in-person surveys were 92.8%, 88.2%, 82.1%, and 87.8%, respectively, at 18 months and at 3, 5, and 10 years.

The SBCSS was approved by the institutional review boards of Vanderbilt University and the Shanghai Municipal Center for Disease Control and Prevention. Written informed consent was obtained from all study participants.

### Outcomes

The primary outcomes of interest were any fracture and osteoporotic fracture that occurred during the 10 years following diagnosis. Osteoporotic fractures were defined as low-trauma fractures (eg, due to falls from standing height), occurring in anatomic sites commonly associated with osteoporosis following the approach of Warriner et al (Supplementary Table 1) (15).

### Study Variables and Covariates Assessment

Menopausal status and age at menopause were assessed at baseline. Women were considered pre-/perimenopausal if they had regular menses or had ceased menstruation for less than 12 months, and menopausal if they had ceased menstruation for 12 months or longer, excluding temporary cessation of menstruation due to pregnancy or breastfeeding.

Information regarding dietary soy intake (consumption of tofu, soy milk, fresh soy beans, and other soy products) was collected at baseline and 18-month surveys using a validated food frequency questionnaire (16). Total soy isoflavone intake was derived by summing the product of soy food intake amount and the isoflavone content of the food item based on the Chinese Food Composition Tables (16,17). Soy isoflavone intake was averaged across the baseline and 18-month surveys to derive a more stable intake assessment. Intake level was further categorized into tertiles in the analysis based on the distribution of the overall cohort: low (<31.38), medium (31.38–56.05), and high (≥56.06 mg/d).

Weight and height were measured following a standard protocol at baseline. BMI was estimated by dividing weight in kilograms by the square of height in meters. BMI was then categorized into underweight (≤18.5), normal weight (18.5–24.9), overweight (25.0–29.9), and obese (≥30.0).

Information regarding exercise was assessed using a validated questionnaire (18). Intensity of exercise was measured by metabolic equivalents (MET) hours per week calculated based on the intensity and duration of a patient’s physical activity (19). MET from the baseline and 18-month assessments were averaged to drive a more stable assessment and further categorized into tertiles of low (<4.5), medium (4.5–12.54), and high (≥12.55) MET hours per week, corresponding to moderate exercise for approximately 1, 1–3, and more than 3 hours per week.

We included tamoxifen in the analysis because it is a SERM with a known influence on bone density and is commonly used in breast cancer treatment. Tamoxifen use (defined as use for greater than 1 month after breast cancer diagnosis) was assessed at baseline and each follow-up encounter. We also calculated cumulative duration of use since cancer diagnosis, which was categorized as 1–16 months and 17 or more months in our analysis.

### Statistical Analysis

Of the 5042 SBCSS participants, we excluded participants with missing fracture data across all three surveys (n = 894) from this study and those with stage IV breast cancer (n = 9) to avoid confounding from bone metastases that could lead to fracture. The remaining 4139 women were included in the fracture incidence analysis: 1987 pre-/perimenopausal and 2152 postmenopausal patients. For the association analyses, we further excluded underweight patients (BMI ≤18.5, n = 120) and patients who had a fracture (n = 19) or had breast cancer metastases (n = 14) within the first 6 months of breast cancer diagnosis because the influence of malnutrition and active cancer treatments is difficult to control for. This yielded 3986 women.

Pearson χ^2^ tests for categorical variables and Student *t* tests for the continuous variables were performed to compare characteristics of patients who developed fracture and those who remained event free. Ten-year fracture incidence rate was estimated by lifetable method.

Multivariable Cox regression models were applied to evaluate the associations of variables under study (ie, soy isoflavone intake, BMI, exercise, and tamoxifen usage) with incidence of any fracture and osteoporotic fracture, reported as hazard ratios (HRs) and 95% confidence intervals (CIs). In the Cox regression, entry time was at the date of enrollment (baseline survey), and exit time was date of first fracture occurrence after cancer diagnosis. Study participants were censored from the analysis at the time of death, self-reported metastases, or last follow-up date, depending on which came first. In the analysis for osteoporotic fracture, patients were censored if they had nonosteoporotic fractures to avoid bias from potential changes in lifestyle and other preventive measures that may be adopted following any type of fracture. Covariates adjusted for in the model included known/suspected risk factors for bone fracture based on the literature and factors that were associated with bone fracture risk in univariate analysis of our own data. These included age at cancer diagnosis, history of bone fracture before cancer diagnosis, use of calcium supplements, parity, education level, aromatase inhibitor use, and breast cancer stage.

Stratified analyses by baseline menopausal status and tamoxifen use evaluated whether these two factors modify the effect of lifestyle factors on bone fracture. Multiplicative interaction was evaluated using the log likelihood ratio test, which compared the model including only the main effects with the model including both main effects and interactive terms. All statistical tests were based on two-tailed probability and a significance level set at alpha (α) less than 0.05.

## Results

The mean age of all patients at baseline was 54.4 years (SD = 10.0). Forty-eight percent of the women were pre-/perimenopausal and 52% were postmenopausal. The 10-year bone fracture incidence was 13.3% for any fracture and 3.6% for osteoporosis fracture; 11.1% and 2.9% for pre-/perimenopausal women and 15.4% and 4.4% for postmenopausal women, respectively. The incidence for osteoporotic fractures by age strata at cancer diagnosis was 2.73% (<50 years), 4.01% (50–59 years), 5.22% (60–69 years), and 3.84% (≥70 years) (Table 1).

**Table 1.** Ten-Year incidence of bone fracture among women with stage 0–III breast cancer

Baseline characteristics	No.	All fractures, incidence rate (%)	Osteoporotic fractures, incidence rate (%)
Overall	4139	552 (13.3)	151 (3.7)
Menopausal status			
Pre-/perimenopausal	1987	220 (11.1)	57 (2.9)
Postmenopausal	2152	332 (15.4)	94 (4.4)
Age, y			
<50	1797	194 (10.8)	49 (2.7)
50–59	1223	171 (14.0)	49 (4.0)
60–69	728	131 (18.0)	38 (5.2)
>70	391	56 (14.3)	15 (3.8)

The mean age of patients with any fractures (55.9 [9.8] years) and osteoporotic fractures (56.2 [9.8 ]years) was older than patients with no fractures (53.6 [10.0] years) at cancer diagnosis. Patients with osteoporotic fractures were more likely to be postmenopausal (*P* = .01) and less likely to exercise (*P* = .03) or to use tamoxifen (*P* = .009). No differences by cancer stage, ER, or progesterone receptor (PR)-positive status and other treatment types were noted across patient groups (Table 2).

**Table 2.** Selected characteristics of study participants in the Shanghai Breast Cancer Survival Study

Characteristics*	No fracture (n = 3484)	Any fracture (n = 502)	*P* †	Osteoporotic fracture‡ (n = 142)	*P*
Age at cancer diagnosis, y	53.6 (10.0)	55.9 (9.8)	<.001	56.2 (9.8)	.005
Age at menopause, y§	49.1 (4.3)	49.2 (4.3)	0.887	49.5 (3.9)	.401
Postmenopause, %	51.2	60.2	<.001	62.7	.012
Age at fracture, y		61.0 (9.8)		61.5 (9.3)	
Smoking, ever, %	2.4	3.8	.063	4.9	.068
Education, %					
<High school	47.5	49.2	.513	55.6	.056
High school	37.3	34.7		27.5	
>High school	15.2	16.1		16.9	
Parity	1.5 (0.9)	1.6 (1.0)	.052	1.6 (1.0)	.066
BMI, %					
Normal weight	62.1	58.2	.087	60.7	.788
Overweight (≥25.0 kg/m^2^)	37.9	41.8		39.4	
Exercise, MET h/wk, %					
<4.50	33.2	35.7	.402	43.0	.030
≥4.50–12.54	33.5	33.9		32.4	
≥12.55	33.3	30.5		24.7	
Soy isoflavone intake, mg/d, %					
Low (<31.38)	33.0	35.1	.530	35.9	.167
Medium (≥31.38–56.05)	33.4	33.7		38.0	
High (≥56.06)	33.6	31.3		26.1	
Cancer stage at diagnosis, %					
≤Stage I	40.2	39.8	.600	40.2	.595
Stage II	51.5	53.2		54.0	
Stage III	8.3	7.0		5.8	
ER, %					
Negative	33.3	34.1	.935	30.3	.054
Positive	65.8	64.9		66.9	
Unknown	0.9	1.0		2.8	
PR, %					
Negative	38.8	44.8	.021	40.9	.208
Positive	60.1	53.6		56.3	
Unknown	1.2	1.6		2.8	
Calcium supp. intake, %	20.3	26.4	.003	22.2	.747
Chemotherapy, %	91.3	89.2	.137	88.7	.332
Radiotherapy, %	30.8	26.9	.077	30.3	.999
Immunotherapy, %‖	15.1	13.0	.214	17.6	.336
Aromatase inhibitors, %	9.4	13.2	.008	14.1	.083
Tamoxifen use, %	57.3	50.6	.004	45.8	.009

* Unless otherwise specified, mean (SD) are presented. BMI = body mass index; ER = estrogen receptor; MET = metabolic equivalent; PR = progesterone receptor.

† *P* values were derived from Pearson’s χ^2^ tests for independence for the categorical variables and Student *t* tests for the continuous variables, both comparing the fracture group of interest with the no fractures group.

‡ Osteoporotic fractures are low-trauma fractures in fragility-associated locations, whereas low-trauma is defined as falls from standing height.

§ Age menopause among postmenopausal women only.

‖ Immunotherapy refers to nonspecific immunotherapy treatments such as interleukin-2 and Interferon.

In the overall study population, soy isoflavone intake was not associated with fracture risk, nor was category of BMI. Exercise of at least 12.55 MET hours per week was associated with reduced risk of osteoporotic fracture, but not for any bone fracture (HR = 0.57, 95% CI = 0.37 to 0.86; and 0.83, 95% CI = 0.66 to 1.03, respectively) compared with exercise less than 4.50 MET. A dose-response association was also observed for osteoporotic fractures (*P*_trend_ = .006). Tamoxifen use was associated with a reduced risk of both any fracture (HR= 0.74, 95% CI = 0.59 to 0.92) and osteoporotic fractures (HR = 0.55, 95% CI = 0.37 to 0.81) (Table 3). A dose-response association was also observed for any fractures and osteoporotic fractures (*P*_trend_ = .002 for both).

**Table 3.** Associations results for bone fracture risk in breast cancer patients

Variables	Any fractures		Osteoporotic fractures	
	No. of events	HR (95% CI)*	No. of events	HR (95% CI)
Soy isoflavone intake, mg/d				
Low (<31.38)	176 / 1327	Reference	51 / 1327	Reference
Medium (≥31.38–56.05)	169 / 1331	1.02 (0.83 to 1.26)	54 / 1331	1.14 (0.78 to 1.68)
High (≥56.06)	157 / 1328	0.93 (0.75 to 1.15)	37 / 1328	0.77 (0.50 to 1.17)
*P*_trend_		.519		.244
BMI				
Normal weight	292 / 2457	Reference	86 / 2457	Reference
Overweight	210 / 1529	1.06 (0.87 to 1.27)	56 / 1529	0.94 (0.66 to 1.32)
Exercise, MET h/wk				
<4.50	179 / 1337	Reference	61 / 1337	Reference
≥4.50–12.54	170 / 1337	0.89 (0.72 to 1.10)	46 / 1337	0.72 (0.49 to 1.06)
≥12.55	153 / 1312	0.83 (0.66 to 1.03)	35 / 1312	0.57 (0.37 to 0.86)
*P*_trend_		.083		.006
Tamoxifen				
Nonuser or user (<1 month)	248 / 1735	Reference	77 / 1735	Reference
Tamoxifen use (≥1 month)	254 / 2251	0.77 (0.64 to 0.91)	65 / 2251	0.63 (0.45 to 0.87)
Duration of tamoxifen use				
<1 month	248 / 1735	Reference	77 / 1735	Reference
1–16 months	143 / 1153	0.86 (0.70 to 1.05)	37 / 1153	0.72 (0.49 to 1.07)
≥17 months	111 / 1098	0.68 (0.54 to 0.85)	28 / 1098	0.54 (0.35 to 0.83)
*P*_trend_		<.001		.003
Among ER+ or PR+ patients				
Tamoxifen				
Nonuser or user (<1 month)	129 / 874	Reference	44 / 874	Reference
Tamoxifen use (≥1 month)	224 / 2027	0.74 (0.59 to 0.92)	57 / 2027	0.55 (0.37 to 0.81)
Duration of tamoxifen use				
<1 month	129 / 874	Reference	44 / 874	Reference
1–16 months	126 / 1006	0.84 (0.66 to 1.08)	32 / 1006	0.64 (0.40 to 1.01)
≥17 months	98 / 1021	0.63 (0.48 to 0.83)	25 / 1021	0.46 (0.28 to 0.75)
*P*_trend_		.001		.002

* HRs and 95% CIs were derived from Cox regression models adjusted for age at diagnosis, education, calcium supplement intake, tamoxifen usage, baseline fracture incidence, parity, aromatase inhibitor usage, and breast cancer stage. BMI = body mass index; CI = confidence interval; ER = estrogen receptor; HR = hazard ratio; MET = metabolic equivalent; PR = progesterone receptor.

Results from stratified analyses by menopausal status are shown in Table 4. Higher soy isoflavone intake was associated with reduced risk of osteoporotic fractures in pre-/perimenopausal but not in postmenopausal women (*P*_interaction_ = .001). Compared with the lowest tertile of soy isoflavone intake (<31.38 mg/d), the highest tertile of soy isoflavone intake (≥56.06 mg/d) was associated with 77% reduced risk of osteoporotic fractures (HR = 0.22, 95% CI = 0.09 to 0.53; *P*_trend_ < .001) in pre-/perimenopausal women. Overweight was associated with an increased risk of osteoporotic fractures (HR = 1.81, 95% CI = 1.04 to 3.14) in pre-/perimenopausal but a marginally reduced risk (HR = 0.67, 95% CI = 0.43 to 1.03) in postmenopausal women (*P*_interaction_ = .01). Exercise was associated with reduced risk of osteoporotic fractures only in postmenopausal women (HR = 0.56, 95% CI = 0.33 to 0.97, for MET hours ≥12.6 vs <4.5) following a dose-response pattern (*P*_trend_ = .035). Tamoxifen use was associated with reduced risk of osteoporotic fracture in pre-/perimenopausal and postmenopausal women, particularly for cases with a long duration of use, although the point estimates did not reach a statistically significant difference, likely because of reduced sample size.

**Table 4.** Associations results for bone fracture risk in breast cancer patients stratified by baseline menopausal status

Variables	Pre-/perimenopausal				Postmenopausal			
	Any fractures		Osteoporotic fractures		Any fractures		Osteoporotic fractures	
	No. of events	HR (95% CI)*	No. of events	HR (95% CI)	No. of events	HR (95% CI)	No. of events	HR (95% CI)
Soy isoflavone intake, mg/d								
Low (<31.38)	75 / 612	Reference	25 / 612	Reference	101 / 715	Reference	26 / 715	Reference
Medium (31.38–56.05)	63 / 647	0.78 (0.56 to 1.09)	22 / 647	0.79 (0.45 to 1.41)	106 / 684	1.22 (0.92 to 1.60)	32 / 684	1.47 (0.87 to 2.48)
High (≥56.06)	62 / 641	0.76 (0.54 to 1.06)	6 / 641	0.22 (0.09 to 0.53)	95 / 687	1.05 (0.79 to 1.39)	31 / 687	1.34 (0.79 to 2.27)
*P*_trend_		.102		<.001		.727		.278
*P*_interaction_						.166		.001
BMI								
Normal weight	124 / 1310	Reference	30 / 1310	Reference	168 / 1147	Reference	56 / 1147	Reference
Overweight	76 / 590	1.36 (1.02 to 1.82)	23 / 590	1.81 (1.04 to 3.14)	134 / 939	0.92 (0.73 to 1.16)	33 / 939	0.67 (0.43 to 1.03)
*P*_interaction_						.042		.010
Exercise, MET h/wk								
<4.50	69 / 665	Reference	25 / 665	Reference	110 / 672	Reference	36 / 672	Reference
≥4.50–12.54	67 / 614	1.02 (0.73 to 1.44)	14 / 614	0.61 (0.32 to 1.19)	103 / 723	0.82 (0.63 to 1.07)	32 / 723	0.79 (0.49 to 1.28)
≥12.55	64 / 621	0.90 (0.64 to 1.27)	14 / 621	0.56 (0.29 to 1.08)	89 / 691	0.76 (0.57 to 1.01)	21 / 691	0.56 (0.33 to 0.96)
*P*_trend_		.556		.071		.053		.035
*P*_interaction_						.366		.980
Tamoxifen								
Nonuser or user (<1 month)	95 / 749	Reference	28 / 749	Reference	153 / 986	Reference	49 / 986	Reference
User (≥1 month)	105 / 1151	0.65 (0.49 to 0.86)	25 / 1151	0.56 (0.32 to 0.97)	149 / 1100	0.84 (0.67 to 1.06)	40 / 1100	0.70 (0.46 to 1.06)
Duration of tamoxifen use								
<1 month	95 / 749	Reference	28 / 749	Reference	153 / 986	Reference	49 / 986	Reference
1–16 months	56 / 559	0.75 (0.53 to 1.04)	12 / 559	0.58 (0.29 to 1.15)	87 / 594	0.92 (0.71 to 1.20)	25 / 594	0.81 (0.50 to 1.33)
≥17 months	49 / 592	0.57 (0.40 to 0.81)	13 / 592	0.55 (0.28 to 1.06)	62 / 506	0.75 (0.56 to 1.01)	15 / 506	0.56 (0.31 to 1.00)
*P*_trend_		.001		.057		.064		.048
*P*_interaction_						.216		.729

* HRs and 95% CIs were derived from Cox regression models adjusted for age at diagnosis, education, calcium supplement intake, tamoxifen usage, baseline fracture incidence, parity, aromatase inhibitor usage, and breast cancer stage. BMI = body mass index; CI = confidence interval; ER = estrogen receptor; HR = hazard ratio; MET = metabolic equivalent; PR = progesterone receptor.

Analyses stratified by both menopausal status and tamoxifen use did not reveal any effect modification (data not shown).

## Discussion

In this large-scale longitudinal study, we found that 13.3% of breast cancer survivors developed a bone fracture and 3.6% had an osteoporotic fracture during the 10-year period following cancer diagnosis. These rates are higher than age-matched healthy women (11.9% for any fracture and 1.4% for osteoporotic fracture, unpublished data), who participated in a population-based cohort study of 75 000 women that we conducted in the same geographic area (The Shanghai Women's Health Study [20]). Observation of increased risk of bone fracture among breast cancer survivors in our study is consistent with previous reports, such as those from the Women’s Health Initiative and the Austrian Breast and Colorectal Cancer Study Group-18 trial (1,21), and was the catalyst for investigating its risk factors in this vulnerable population.

We found the first evidence that high soy isoflavone intake was associated with a low risk for osteoporotic fractures in pre-/perimenopausal but not in postmenopausal breast cancer survivors. Soy isoflavones, a natural SERM, have been shown to be inversely associated with risk of recurrence and death in breast cancer patients (2,22). The association of soy isoflavone intake with osteoporotic fractures, however, has been disputed. Several epidemiologic studies showed that soy food consumption was related to a reduced risk of osteoporotic fracture in postmenopausal women, including a prospective study we conducted in the same region among general Chinese women (The Shanghai Women's Health Study) in which we found the association was primarily seen in women who recently became menopausal (8). However, a 2009 meta-analysis of 10 randomized controlled trails concluded that a mean dose of 87 mg/d isoflavone supplement for 1 year, a dose within the highest tertile for our study, did not affect BMD in postmenopausal women (23). On the other hand, a recent review of randomized controlled trials suggested that soy isoflavone consumption during the menopausal transition might prevent reduction in BMD and promote bone health (24), suggesting that influence of soy isoflavone intake on bone loss may depend on the timing of exposure (25). Our finding of no association among postmenopausal breast cancer survivors and an inverse association among pre-/perimenopausal patients is consistent with findings of clinical trials on soy isoflavone consumption and BMD during the menopausal transition. Although tamoxifen and soy isoflavones are both SERMs and may compete to bind the same ERs, we observed no effect modification by tamoxifen on soy-fracture association in pre-/perimenopausal and postmenopausal women.

We observed that overweight/obesity was associated with increased relative risk of osteoporotic fractures among premenopausal breast cancer patients but a statistically nonsignificantly reduced risk among postmenopausal women. The latter may be attributable, in part, to the low number of obese patients included in the present study. Previous studies, including two recent meta-studies (10,12), have linked higher BMI with reduced risk for overall osteoporotic fractures among postmenopausal women, an association attributable to the fact that fat tissue is the primary site of estrogen production in postmenopausal women (3). In premenopausal women, reduced fitness, circulating vitamin D level (26), and increased inflammation related to obesity (27) may explain our observed increased bone fracture risk. It is noteworthy that premenopausal women were underrepresented in previous studies, and our study is the first to our knowledge to include such a large number of pre-/perimenopausal breast cancer survivors for research on osteoporotic fracture. Thus, our findings need to be replicated in future studies.

Consistent with previous studies showing that physical activity is associated with reduced risk of osteoporotic fractures in older women (11,13), we found that exercise was associated with a reduced risk among breast cancer survivors following a dose-response pattern. Exercise may enhance coordination, balance, mobility, and muscle strength, helping reduce the likelihood of falls that may cause fractures (11). Weight-bearing exercise has also been shown to prevent loss of BMD, which likely contributes to a reduced risk of osteoporotic fractures (28).

As expected and consistent with several previous studies, including a large population-based study reporting 32% reduced risk of osteoporotic fracture for tamoxifen use (29), we found that tamoxifen use was associated with a 37% reduced risk of osteoporotic fractures among breast cancer survivors. This association was not modified by menopausal status. Tamoxifen, a SERM that acts as an estrogen agonist in the bone, has been shown to preserve BMD in postmenopausal women (30,31), an established predictor for osteoporotic fracture risk (32). However, in premenopausal tamoxifen users, it was suggested that the partial agonist effect in the bone may not to be strong enough to compensate for reduction of estrogen effect, leading to loss of BMD, particularly among those who are amenorrhoeic (33). In line with this, a 2006 study of 159 Finnish premenopausal breast cancer patients showed that tamoxifen use was associated with increased BMD in premenopausal patients who developed treatment-induced early menopause but was related to decreased BMD among those who remained premenopausal (33). About two-thirds of pre-/perimenopausal breast cancer patients in our study entered menopause within 2 years after cancer diagnosis, which may explain the inverse tamoxifen association we observed in pre-/perimenopausal and postmenopausal survivors.

To our knowledge, this is the first study to evaluate the association of soy food consumption with osteoporotic bone fracture among breast cancer survivors and the largest investigation on other modifiable risk factors. The strengths of this study include the longitudinal study design, repeated exposure measurements, and high response rate. Some limitations, however, exist. First, fracture incidences and exposures were self-reported. Therefore, misclassification, particularly for osteoporotic fractures, is possible. However, a previous study has shown self-reported fracture incidences were, in general, accurate (34). Our finding on tamoxifen and bone fracture provides support toward the validity of self-reported outcome. Our repeated exposure assessments should have reduced measurement errors in exposure assessments. Second, although we adjusted for aromatase inhibitor use (prevalence rate ranged from 9.36% to 14.08% in patients without and with a bone fracture) in our study, information on osteoporosis screening, bisphosphonate use, and compliance with concurrent guidelines for osteoporosis prevention was unavailable, which may bias our findings. However, it is unlikely that many patients were treated with bisphosphonates in our study because guidelines for osteoporosis screening, prevention, and treatment for breast cancer patients did not exist in China until 2013 and were finalized in 2015 (35). Because all our study participants were diagnosed and enrolled between 2002 and 2006, most of our study participants were already 8 to 10 years post-cancer diagnosis by 2015. Thus, the influence of bisphosphonate therapy on our study results is expected to be minimal. Finally, the treatment regimens of patients included in our study also reflect the standard practice during the study period. Treatment guidelines for breast cancer and fracture prevention have both evolved over the last decade. Thus, some of the findings may not be generalizable to women with breast cancer receiving contemporary treatment regimens that may include concurrent bisphosphonate therapy for postmenopausal women or prophylaxis for patients who are at increased fracture risk based on fracture risk algorithms. However, compliance with fracture risk assessment and management guidelines is not always optimal in many countries, including China (36). Finally, it is well-established that nonpharmacologic prevention approaches form the underpinnings of osteoporosis prevention and self-management strategies, and patients should be educated in these approaches even when considering pharmacologic therapy.

In summary, we found novel evidence that among pre-/perimenopausal breast cancer survivors, soy isoflavone intake is inversely associated with osteoporotic fracture risk, and overweight/obesity with increased osteoporotic fracture risk. Exercise and tamoxifen use were inversely associated with the risk for osteoporotic fracture. Our findings, if confirmed, can help guide development of comprehensive fracture risk reduction strategies (eg, through patient screening, diagnosis, treatment, and self-care approaches) in this vulnerable population.

## Funding

The SBCSS was supported by grants from the Department of Defense Breast Cancer Research Program (DAMD 17-02-1-0607) and the National Cancer Institute (R01 CA118229). The funding organizations had no role in the design and conduct of the study; collection, management, analysis, and interpretation of the data; or preparation and approval of the manuscript. The content of the information does not necessarily reflect the position or the policy of the government, and no official endorsement should be inferred.

## Notes

Affiliations of authors: Yale College, Yale University, New Haven, CT (NZ); Section of Rheumatology, Yale School of Medicine, Yale University, New Haven, CT (EH); Division of Epidemiology, Department of Medicine, Vanderbilt-Ingram Cancer Center, Vanderbilt University Medical Center, Nashville, TN (HC, XOS); Shanghai Municipal Center for Disease Prevention and Control, Shanghai, China (LS, KG, YZ, PPB); Shanghai Cancer Hospital, Fudan University, Shanghai, China (YZ).

The authors declare no conflicts of interest regarding the publication of this article.

The authors are in debt to the study participants and research team of the SBCSS. Dr Hsieh is supported by NIH/Fogarty International Center K01TW009995 and the Yale Center for Clinical Investigation/Doris Duke Foundation Fund to Retain Clinical Scientists.

## Supplementary Material

Supplementary_Data_pkz017
