# Supplementary material for: Soy Food Consumption, Exercise, and Body Mass Index and Osteoporotic Fracture Risk Among Breast Cancer Survivors: The Shanghai Breast Cancer Survival Study
Source: JNCI Cancer Spectr. 2019 May 21;3(2):pkz017. doi: 10.1093/jncics/pkz017 (PMC6527440; doi:10.1093/jncics/pkz017)
Supplement: Supplementary_Data_pkz017 [file supplementary_data_pkz017.pdf]

**Supplementary Table 1.** Fracture Sites

| Osteoporotic Fracture Sites [a] | Other Fracture Sites |
|---|---|
| Lumbar spine | Skull |
| Rib | Clavicle |
| Humerus | Palm |
| Elbow | Finger |
| Forearm (radius or ulna) | Foot |
| Wrist | Toe |
| Sacrum | |
| Pelvis | |
| Hip | |
| Femur | |
| Patella | |
| Tibia | |
| Ankle | |
| Calcaneus | |

[a] Fractures caused by high impact, such as trauma, were excluded
